# Patient-reported factors that influence the vestibular schwannoma treatment decision: a qualitative study

**DOI:** 10.1007/s00405-020-06401-0

**Published:** 2020-10-07

**Authors:** O. M. Neve, G. Soulier, M. Hendriksma, A. G. L. van der Mey, A. van Linge, P. P. G. van Benthem, E. F. Hensen, A. M. Stiggelbout

**Affiliations:** 1 Department of Otorhinolaryngology and Head and Neck Surgery, Leiden University Medical Center, P.O. Box 9600, 2300 RC Leiden, The Netherlands; 2 Department of Otorhinolaryngology and Head and Neck Surgery, Erasmus Medical Center, Rotterdam, The Netherlands; 3 Medical Decision Making, Department of Biomedical Data Sciences, Leiden University Medical Center, Leiden, The Netherlands

**Keywords:** Vestibular schwannoma, Shared decision-making, Qualitative research, Patient-centered care

## Abstract

**Purpose:**

In cases of small- to medium-sized vestibular schwannomas, three management strategies can be opted for: active surveillance, surgery or radiotherapy. In these cases, the patient’s preference is pivotal in decision-making. The aim of this study was to identify factors that influence a patient’s decision for a particular management strategy.

**Methods:**

A qualitative inductive thematic analysis was performed based on semi-structured interviews. Eighteen patients with small- to medium-sized vestibular schwannomas were interviewed. All patients were diagnosed or treated at one of the two participating university medical centers in the Netherlands.

**Results:**

Ten themes were identified that influenced the decision, classified as either medical or patient-related. The medical themes that emerged were: tumor characteristics, the physician’s recommendation, treatment outcomes and the perceived center’s experience. The patient-related themes were: personal characteristics, anxiety, experiences, cognitions, logistics and trust in the physician.

**Conclusion:**

Knowledge of the factors that influence decision-making helps physicians to tailor their consultations to arrive at a true shared decision on vestibular schwannoma management.

## Introduction

Vestibular schwannoma (VS) is a benign intracranial tumor, arising from schwann cells of the vestibular branch of the vestibulocochlear nerve. Current management options for VS generally consist of one of three modalities: microsurgery, radiotherapy or active surveillance (also known as wait-and-scan policy) [1]. Based on the available evidence, a clinical equipoise exists in the management of small- to medium-sized VS (up to 25 mm in extrameatal diameter). Active surveillance is a valid management option, especially in non-progressing tumors. However, hearing loss and vestibular problems may increase, even in otherwise stable tumors. In patients with (progressing) medium-sized tumors, both surgery and radiotherapy are viable options with equally high tumor control rates and generally good facial nerve outcome. Long-term hearing results are universally poor and vestibular function may be impacted, both after surgery and radiotherapy. However, both modalities differ considerably in their mode of action, administration and the way eventual sequelae and side effects become apparent, either suddenly (i.e., after surgery) or after a time interval (i.e., radiotherapy). The advantages and disadvantages of both modalities need to be weighed and patient and physician can explore treatment options together, a process that is also known as shared decision-making (SDM). In case there is no clear superiority of one modality over the other from a medical point of view, patient preferences are important. SDM has been argued to be the preferred model in preference sensitive decisions, as in small- to medium-sized VS management [2–4]. A four-step model is often used to apply SDM in clinical practice [4]. First, the physician informs the patient that a decision is to be made and that the patient’s opinion is important. Second, the pros and cons of each relevant option are explained. Next, the physician and patient discuss the patient’s preferences and the physician supports the patient in deliberation. Finally, the patient’s decisional role and preference are discussed and the decision is made or deferred. To make this process work satisfactorily, an understanding of the factors that influence patients’ decision are essential.

Known medical factors influencing decision-making in VS are tumor size, tumor progression, symptoms, risk of complications and the physician’s recommendation [5]. Practice variation may arise if patients do not receive unbiased information about all possible treatment options [5, 6]. Patient factors that influence the VS treatment decision are less well known. As of yet, the only identified factors that influence decision-making are anxiety and logistics [7, 8]. For various other diseases, it has been reported that patient’s coping and decision-making style are also influential in the clinical decision. However, these factors have not been reported in VS patients [9, 10]. This qualitative study aims to identify factors that influence a patient’s decision.

## Materials and methods

### Design

A qualitative interview study was performed, followed by an inductive thematic analysis. Qualitative research allows exploring the notions of the respondents, without directing the answers by predefined questions or answering categories, as is the case in quantitative research. It can provide rich data and new insights about patient-reported factors of importance for treatment decisions [11]. Using this thematic qualitative analysis, patterns within the data were identified, analyzed and reported. Methods and results are reported in accordance with Standards of Reporting Qualitative Research [12].

### Ethics

The Medical Ethics Committee of the Leiden University Medical Center reviewed the protocol (P16.064) and concluded that their approval was not required under Dutch law.

### Recruitment

Purposive sampling was used to enroll patients with medium-sized VS (i.e., 10–25 mm extrameatal diameter) from two tertiary care centers in the Netherlands, Erasmus Medical Center (EMC) and Leiden University Medical Center (LUMC). Both centers are experienced in the treatment of VS and offer surgery (mostly the translabyrinthine or retro sigmoid approach) and stereotactic fractionated (LUMC) or single dose (EMC) radiotherapy. Ambulatory procedures are similar, consisting of an initial consultation with an otorhinolaryngologist, followed by a multidisciplinary team (MDT) meeting attended by an otorhinolaryngologist, a neurosurgeon and a radiation oncologist and a subsequent consultation to discuss treatment options. Potential participants were identified from the records of the weekly MDT meeting in which all new patients with a VS are discussed. Patients were provided with information about the study by their treating physician during the subsequent consultation. One of the researchers (GS) followed up with a phone call after 1 week to check the willingness to participate.

### Interviews

Semi-structured, face-to-face interviews were conducted to gather nuanced and context-dependent data [13]. The interviews were carried out using a topic guide, an outline of key issues and areas to explore during the interview (Table 1). This form of interviewing allows for new ideas to be brought up during the interview and to be incorporated in subsequent interviews. Participants were interviewed at a location of their choice (generally their own home). Conversations typically lasted between 30 and 90 min and were audio recorded. All interviews were conducted in Dutch and by the same interviewer (GS), who was not involved in patient care during this period. Interviewing was carried out until data saturation occurred, which was defined as the point when no new ideas emerged from the interviews. To this aim, data analysis was carried out concurrently. The stage at which data saturation occurred was determined by consensus within the research team (GS, MH and ON).

**Table 1:** Topic guide used for semi-structured interviews

Topic guide interviews
Contextual information
Information provision
People of influence
Aim of treatment
Decision-making process
Priorities in decision-making
Barriers in decision-making

### Analysis

All interviews were transcribed verbatim and imported in the qualitative analysis software ATLAS.ti (ATLAS.ti, version 8.4.18, GmbH; Berlin, Germany). Data were analyzed using the framework method. This method uses a framework matrix for data interpretation by charting in rows (patients) and columns (codes). This provides a structure into which data can be systematically reduced, facilitating analysis [14, 15]. Data were coded with open codes that emerged from the text. Codes were initially assigned by one researcher (for the first eight interviews by GS and the other ten by ON). During the coding, both researchers met regularly with another member of the research team (MH) to review the codebook and discuss the interpretation of data. Coding was reviewed in ten interviews by a third researcher (MH). All research team members are medical doctors (MD). ON is a medical doctor and researcher trained in coding and analyzing patient interviews. At the time of the study, MH and GS were involved in patient care as trainee specialists. However, none of the researchers that conducted or analyzed the interviews (MH, GS or ON) were directly involved in in the care of the study patients.

## Results

Data saturation was reached after 18 interviews. Nine patients from each center were included, one patient visited both centers. In eight interviews, a spouse or a relative was present.

Patients experienced the decision-making as challenging, because it was hard to weigh the advantages and disadvantages of the treatment modalities.

“It is a choice between… Actually, between three evils. There is nothing good. None of the three is good, because all have their consequences” patient 2.

Decision-making styles varied among the patients. Some patients indicated to defer decisions generally, this strategy usually resulted in a preference for active surveillance. Other patients were more decisive and had even decided before the first consultation.

“I can still postpone [the decision] a little, so I don’t let it get to me any more than I need to at the moment” patient 9.

This difference in patients’ decision-making style was also reflected in the search for information on the disease and the treatment modalities. Some patients refused to search for information on the internet because of the fear of finding the worst case stories. A majority of the patients looked for information on websites of hospitals and patient associations. Others also looked for patient’s experiences on social media. A minority searched in medical literature.

**Fig. 1:**
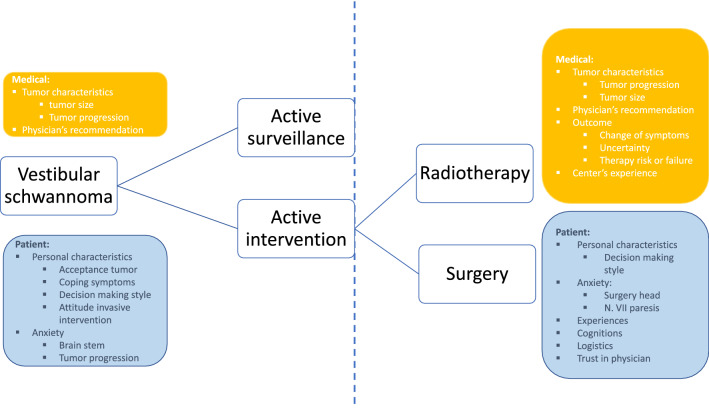
Two-step decision model based on the patient's experiences with factors that influence the decision-making

Although from a medical point of view, multiple management options and timing of possible interventions make the clinical decision complex, patients expressed that they experienced a rather straightforward two-step decisional process, as shown in Fig. 1. First, the decision between active surveillance and active treatment had to be made. Second, when active treatment was chosen, the patient had to decide between surgery and radiotherapy. Each decision was influenced by different factors, although a few factors influenced both decisions. We classified all factors as either medical or patient-related. Medical factors were defined as factors related to the tumor, physician or the treatment modality and patient factors were related to personal characteristics, experiences or cognitions.

### Active surveillance vs. active treatment

The medical factors that patients perceived to have influenced the first decisions were tumor characteristics and physician’s recommendation. Tumor characteristics, such as tumor size and progression, were important to determine whether active surveillance was still considered an option. Conversely, in the absence of tumor progression, active surveillance was generally deemed preferable.

“When the tumor is growing, surgery should be performed” patient 11.

In addition, the physician’s recommendation affected decision-making. Most patients acknowledged the authority of physicians because of their knowledge and experience.

“Physicians have influence on the decision. Despite all information on the internet nowadays, I rely on their know-how, they know what they are talking about, that is really important” patient 5.

Several patients were content when physicians offered all treatment options without a recommendation; however, some wanted more guidance from the physician. When no recommendation was given, these patients looked for clues as to the physician’s own treatment preference. Some of them were surprised and sometimes disappointed when physicians did not provide any treatment advice. The majority of the patients, however, stated that in the end, they felt that the decision was theirs to make, regardless of their need for guidance by their physician.

The decision between active surveillance or active treatment was influenced by two patient factors: anxiety and personal characteristics. Anxiety, specifically about tumor progression and brainstem compression, prompted patients to choose active treatment, all the more so if patients were surprised by the close anatomical relation between the tumor and the brainstem.

“It came as a shock … So, it [the tumor] is pushing against the brainstem.” patient 7.

Personal characteristics included coping with symptoms, tumor acceptance, attitudes toward invasive treatment and decision-making style. Patients that were less troubled by their symptoms tended to prefer active surveillance. Some patients preferred avoiding medical interventions in general and thus favored active surveillance. In contrast, some patients experienced difficulty with the concept of a persisting tumor inside their head. This lack of tumor acceptance made patients choose an active treatment.

“I want it [the tumor] to be removed! Surgery. Get rid of it.” patient 3.

### Surgery vs. radiotherapy

Medical factors influencing the decision between surgery and radiotherapy were treatment outcome and a perceived center’s experience with the treatment. In addition, this decision was also influenced by tumor characteristics and physician’s recommendation. Treatment outcome contained three components: change of symptoms, uncertainty about tumor control and therapy risk or failure. Uncertainty about the occurrence of complications made the decision complex, because patients found it hard to translate the group-based probabilities to their individual situation.

The perceived possibility of a change in symptoms or symptom severity as a result of therapy, such as the possibility of improvement of vestibular or trigeminal symptoms after surgery also affected patients’ choices. Patients were generally aware that neither treatment modality improves hearing or facial paresis. Uncertainty about tumor control and therapy-associated complications influenced the patients’ decision.

“At first, I looked whether there was a treatment that could improve my symptoms, there isn’t. Then, I looked at the least risky treatment.” patient 3.

Uncertainty about results after radiotherapy was caused by the lengthy time interval between the treatment and its effects or its complications. Because of this uncertainty, some patients preferred surgery, after which the effects and complications are immediately apparent.

“What also was of influence, is that we understood that radiotherapy… You never know immediately whether it was effective, it can take a year, in the worst case, to notice the effect or to notice that it has done nothing.” Patient 9.

A proportion of the patients mentioned that the possible consequences of treatment failure influenced their decision-making. Treatment failure was defined as tumor progression after initial treatment that necessitated additional active treatment. When surgery had been performed as initial therapy, radiotherapy is the additional treatment of choice, if necessary and vice versa. Patients sometimes preferred surgery as the initial treatment because they felt or were informed that surgery after radiotherapy failure could be more challenging due to fibrotic tissue.

“In the future, potential recurrent tumors cannot be treated or at least not as well [after radiotherapy]” patient 4.

The last medical factor of influence was the perceived center’s experience with radiotherapy or surgery. Patients generally defined experience by the number of procedures yearly performed at the center. A number of patients directly enquired about the center’s experience in VS surgery and radiotherapy and considered the perceived experience in their decision.

“They have the most experience. When you have to undergo such complex surgery, you want the best team there is” patient 13.

We identified several patient factors influencing the decision between surgery and radiotherapy: the patients’ cognitions, the patients’ experience, logistics and trust in the physician. In addition, anxiety played a role in the decision between surgery and radiotherapy. Anxiety about facial paresis specifically was reported by most patients.

“The neurosurgeon could not guarantee that no facial paresis would occur. In case that it would happen, it would be terrible.” patient 1.

The anxiety about facial paresis did not differentiate between the two options because the perceived preservation of facial function was comparable. However, some patients favored surgery because they thought that surgical recovery of the facial nerve was possible only if the paresis was caused by surgery.

Other patients were afraid of surgical procedures inside the head and therefore favored radiotherapy. This anxiety was closely linked to other cognitions about the treatment. Some patients thought that radiotherapy was less invasive and, therefore, safer than surgery or, conversely, that radiotherapy did not solve anything. In addition, patients were influenced by their own or others’ previous experiences with radiotherapy or surgery.

“I was thinking, opening my head and messing around, that was in my opinion not such a good idea” patient 3.

“because in my opinion, radiation does not solve anything” patient 6.

Logistics was also a factor that influenced decision-making. The perceived time investment associated with either surgery or radiotherapy and the perceived impact on work, study, normal daily life or holidays affected the decision between surgery and radiotherapy as well as the decision on timing of the start of treatment.

“The radiotherapy that is something I need to think about, every day traveling to the hospital and back is something I do not like” patient 12.

Trust in physicians was the final factor that influenced decision-making. Trusting the physician’s capabilities and expertise was a prerequisite for choosing either surgery or radiotherapy. In addition, patients wanted to attain some level of affinity with their treating physician. A lack of trust, consisting of both confidence and affinity, made patients want a second opinion.

“They are the experts, but there should also be some connection, some human touch” spouse of patient 13.

## Discussion

This qualitative study identified patient-reported factors that influence decisions in VS management. The decision-making process entails one or two steps; the first step comprises the decision between active surveillance and active treatment. When active treatment is opted for, a second decision between the two active treatment modalities, radiotherapy or surgery, ensues. Both steps were influenced by factors that could be classified as medical or patient-related. Medical factors were tumor characteristics, physician’s recommendation, treatment outcomes and center’s experience with the treatment. Patient-related factors were anxiety, personal characteristics, experience, cognitions, logistics and trust in physician.

Qualitative research enables researchers to find explanations for observations using the diversity of data and does not aim to provide generally transferable data. Although data saturation was reached, other themes may arise in different clinical or cultural contexts. Another challenge in qualitative research is to minimize the influence of the researcher’s own preferences and assumptions. This is partly ensured by the use of the clear and transparent framework approach and the use of multiple coders, none of whom were directly involved in the patient care pathway.

Several medical factors have previously been described in quantitative research on VS. The physician’s recommendation has been reported as the most influential factor in a patient’s decision [5, 6, 16, 17]. Tumor characteristics have been described as an additional influencing factor both in the decision between surgery and active surveillance and in the decision between treatment modalities [5, 16]. Treatment outcomes such as change of symptoms and tumor control have also been reported as influencing factors in several studies, both quantitative and qualitative [6, 7, 17]. Our study added the theme of treatment failure, an important determinant.

Patient-related factors on VS decision-making that have been previously reported are anxiety and logistics [7, 8]. An important aspect of the factor anxiety identified in our study is the perceived risk of facial paresis. This is in line with a study of Müller et al., reporting that patients ranked facial paresis as the most severe sequela [6]. Another aspect of anxiety is the perceived risk of complications of treatment. This was also reported in the qualitative study of Linkov et al. [7]. The latter study also identified doubts about making the right decision as a factor, but this was not corroborated in the current study. Only one aspect of the factor logistics, i.e., return to work, has been previously identified in a decision trade-off study [8].

Other patient-related factors identified in this study have not been previously reported for VS, but have been investigated in other diseases. For example, personal characteristics, such as decision-making style and a patient’s trust in the physician have been reported to influence treatment decisions in metastatic breast cancer [18]. The patient’s cognitions about therapy and their own past experiences have been shown to influence management decisions in diabetes mellitus type II and lumbar disc herniation [19, 20]. The patient’s coping abilities and level of disease acceptance have been reported to affect treatment decisions in recurrent prostate cancer [21].

Truly shared decision-making requires the adequate and unbiased information of patients [4]. In addition to information provided by physicians, patients searched the internet for information about the disease, the treatment and experiences of other patients. The use of internet by otorhinolaryngology patients has increased over the years and has an increasing clinical impact [22]. However, the quality of online VS information varies highly [23]. It is important that physicians explore any preconceptions that a patient might have about the disease and the relevant treatment options to tailor the clinical information to the patients level of knowledge, deal with misconceptions if present and to ensure that patients fully understand the pros and cons of the treatment options.

### Implications for practice

The findings of this study can be used to improve information and care provision in daily practice. In this study, one of the important medical factors that influenced the decision-making was physician’s recommendation. The physician’s medical specialty will probably influence the provided recommendations, i.e., surgeons tend to advise surgery more often, whereas radiation oncologists tend to advise radiotherapy [5, 6]. This could lead to unwanted practice variation. Moreover, the patients’ cognitions about treatment, which were not always correct, also impacted the treatment decision. To overcome these problems, patients with small- to medium-sized VSs should be informed about all viable treatment options, preferably by all specialties involved in the different management strategies (radiotherapy, otorhinolaryngology and/or neurosurgery). This seems the best way to ensure balanced information on which patients base their decision.

In addition, the information provided during consultations could be better adapted to patient-related factors that influence decision-making. Personal characteristics, cognitions and anxieties and their influence on the treatment decision can be addressed, but only if the physician is able to identify these factors.

Lastly, physicians should be aware that patients are also influenced by medically irrelevant factors such as accessibility of care, required time investment or even holiday planning.

Tailoring information provision to an individual patients’ needs could enhance patient involvement in clinical decision-making, which has been shown to reduce decisional conflict in VS patients [24].

These insights into the factors that influence the patients’ decision can be used to improve the decision-making process in a number of ways. First, patients with an indication for active treatment, in whom radiotherapy and surgery are both viable treatment options according to the MDT meeting, should be informed about both treatment modalities. To ensure balanced information about the effectiveness and downsides of radiotherapy and surgery, it is now provided by both a radiation oncologist and a surgeon (either a neurosurgeon or otorhinolaryngologist) in sequentially planned consultations at one of the participating centers. Second, patient-related factors could be better identified and monitored using patient-reported outcome measures (PROMs) structurally. The factors that influence the patients’clinical decision as identified in this qualitative study can thus subsequently be evaluated in a quantitive way, to study their prevalence and relative importance. Anxiety, for example, is identified by the anxiety subscale of a disease-specific quality of life questionnaire, the Penn Acoustic Neuroma Quality of Life (PANQOL) [25]. In addition, to help patients to cope with their anxiety a psychologist has been added to the VS care team. Third, public information on hospital websites and patient information flyers could be improved by involving patient representatives to better align the information with the patients’ needs and expectations.

### Conclusion

This study provides new insights into the factors that influence patients’ decision-making in small- to medium-sized VSs. Medical factors, such as tumor characteristics and the physician’s recommendation were confirmed to play a role. In addition, new patient-related factors were identified, such as decision-making style, the patients’ trust in the physician, the patient’s cognitions about therapy and past experiences and the patient's personal characteristics. Awareness of these factors is important for adequate patient counseling and may help in reaching truly shared VS management decisions.
